# The effectiveness of blinatumomab in clearing measurable residual disease in pediatric B‐cell acute lymphoblastic leukemia patients detected by next‐generation sequencing

**DOI:** 10.1002/cam4.6771

**Published:** 2023-12-08

**Authors:** Min'er Gu, Yahong Xia, Jingying Zhang, Yongmin Tang, Weiqun Xu, Hua Song, Xiaojun Xu

**Affiliations:** ^1^ Division of Hematology‐Oncology Children's Hospital of Zhejiang University School of Medicine, The Pediatric Leukemia Diagnostic and Therapeutic Technology Research Center of Zhejiang Province, National Clinical Research Center for Child Health Hangzhou China

**Keywords:** acute lymphoblastic leukemia, blinatumomab, measurable residual disease, next‐generation sequencing, pediatric

## Abstract

**Background:**

Blinatumomab improved survival outcomes in B‐cell acute lymphoblastic leukemia (B‐ALL) patients with measurable residual disease (MRD) <10^−4^. However, data on blinatumomab clearing MRD with high sensitivity of 10^−6^ remain scarce. This study evaluates the effectiveness of blinatumomab in eradicating extremely low level (up to <10^−6^) of MRD, as detected by next‐generation sequencing (NGS), in children with B‐ALL.

**Methods:**

Patients (*n* = 19) whose MRD was undetectable by multiparameter flow cytometry (MFC) (sensitivity of 10^−4^) but detectable by NGS after chemotherapy and followed by blinatumomab consolidation were included retrospectively.

**Results:**

After one course of blinatumomab, 13/19 patients (68%) successfully achieved NGS‐MRD clearance (undetectable). With a median follow‐up of 13.3 months, three of patients who were NGS‐MRD positive relapsed within 1.8 months, while another three remained complete remission.

**Conclusions:**

Our study was the first to demonstrate that blinatumomab could further eradicate MRD after patients achieve MFC‐MRD undetectable in B‐ALL patients.

## Graphical abstract

This study was the first to demonstrate that blinatumomab could further eradicate MRD after patients achieve MFC‐MRD undetectable in B‐ALL patients. With the development of novel and more sensitive MRD detection techniques and advent of new immunotherapies such as blinatumomab, targeting a deeper molecular remission in B‐ALL has now become a reality.

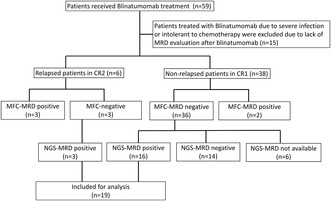

## 1 INTRODUCTION

Measurable residual disease (MRD) is a strong prognostic factor in B‐cell precursor acute lymphoblastic leukemia (B‐ALL). Blinatumomab, a CD3/CD19‐directed bispecific T‐cell engager (BiTE®), induces high rates of complete MRD response by multiparameter flow cytometry (MFC) or reverse transcriptase‐polymerase chain reaction (PCR) in B‐ALL patients.^1^ In a phase III study of children with high‐risk first‐relapsed B‐ALL, blinatumomab consolidation resulted in improved MRD remission (<10^−4^) and event‐free survival (EFS) compared to chemotherapy, independent of baseline MRD.^2^ Among patients with MRD remission, a higher proportion of those treated with blinatumomab had undetectable MRD compared to patients treated with chemotherapy, suggesting that blinatumomab induces deeper responses than that measured with a 10^−4^ MRD sensitivity level.^2^ In pediatric ALL, next‐generation sequencing (NGS) is increasingly being used in clinical practice to comprehensively define genetic alterations and to enhance the sensitivity of MRD detection compared with current methods. The NGS‐MRD approach has been developed to detect MRD at a sensitivity of 1 × 10^−6^.^3^ It has been shown that the NGS assay can detect very low levels of MRD in patients who were considered “MRD undetectable” by less sensitive MFC or PCR technologies.^4^, ^5^ Early achievement of undetectable NGS‐MRD identifies patients who have a very low risk of relapse. However, there is limited data available on NGS‐MRD in blinatumomab therapy for B‐ALL. We thus retrospectively reviewed the clinical utility of NGS assay in pediatric patients with B‐ALL treated with blinatumomab and demonstrated drug's efficacy in eradicating MRD after patients achieve undetectable MFC‐MRD.

## 2 METHODS

Between October 2021 and February 2023, 19 pediatric B‐ALL patients fulfilling the following criteria were included in this retrospective study: (1) presenting undetectable MFC‐MRD but positive NGS‐MRD and treated with blinatumomab due to the remaining NGS‐MRD; (2) For newly diagnosed patients, they finished induction treatment and remained CR1; For relapsed patients, they were in CR2; (3) The informed consent on blinatumomab treatment to eradicate NGS‐MRD was obtained from the parents. The details are shown in Figure S1. For newly diagnosed patients, we monitored MFC and NGS MRD together at the interim of induction (3th week), end of induction (5th week), and end of consolidation (about 12th week). For the following timepoints after end of consolidation, MFC‐MRD was determined every 2–3 months, and the determination of NGS‐MRD was done according to the parents' requirements. For patients receiving blinatumomab, NGS‐MRD was determined before and at the end of blinatumomab administration. The monitoring of NGS‐MRD after blinatumomab proceeded according to the parents' requirements.

Next‐generation sequencing analysis for the identification and sequential monitoring of clonal Ig rearrangements was performed by high‐throughput deep‐level sequencing using the Seq‐MRD® (ImmuQuad Biotech) as previously described.^6^

For this, the genomic DNA was extracted from bone marrow samples using QIAamp DNA Mini kit (Qiagen). The amplified and purified DNA was sequenced using the Illumina® Novaseq PE150 (Illumina® Novaseq) platform through which the sequences and frequencies of the different clonotypes in the samples were obtained. The frequency of each clonotype in a sample was determined by calculating the number of sequencing reads for each clonotype divided by the total number of passed sequencing reads in the sample. The sequences of IGH‐VDJ, IGH‐DJ, IGK, and IGL were assessed for clonality with dominant index sequence(s) defined as meeting all the three following criteria: (1) ≥3% frequency within all the recombined molecules of the assay, (2) ≥0.02% frequency within total input MNC (as determined by total input DNA), and (3) sufficient separation in the distribution of each trackable sequence with no obvious regularity between sequences.

## 3 RESULTS

Baseline patient characteristics are shown in Table 1. Median age at diagnosis was 4.4 years (range 0.3–10.6), with males accounting for 84% of patients. In total, 71 clonal rearrangements were detected by NGS, including 27 IGH clonal rearrangements in 15 (79%) patients, 9 IGH‐DJ in 6 (32%), 14 IGK in 8 (42%), 6 IGKDE in 9 (32%), and 12 IGL in 8 (42%) patients.

**TABLE 1:** Patients' characteristics, NGS‐MRD, and outcome.

ID	Age at Dx (years)	Sex	Risk status at Dx	Fusion genes	Time of blina since diagnosis or relapse (months)	Disease status	Pre‐blina NGS‐MRD	Pre‐blina positive clones	Post‐blina NGS‐MRD	Post‐blina positive clones	Relapse after the blina	HSCT (months after blina)	Status at last follow‐up (months after blina)
Pt#1	10.3	M	LR		3.5M	CR2	Positive (0.92%)	IGH1 and IGH2	Undetectable		No	Yes (2M)	Live/CR (21.7M)
Pt#2	0.8	M	HR	MLL‐AF10	4.1M	CR1	Positive (0.707%)	IGH1, IGH2, and IGL1	Positive (63.385%)	IGH1, IGH2, and IGL1	Yes, one week after blina, both bone marrow and EM relapse	Yes (3M)	Live/CR (16.7M)
Pt#3	6.4	M	LR	TEL‐AML1	2.3M	CR1	Positive (0.003%)	IGH2 and IGKDE3	Undetectable		No	No	Live/CR (15.9M)
Pt#4	5.3	M	HR		2.3M	CR1	Positive (0.922%)	IGH1, IGH2, IGH3, and IGKDE1	Undetectable		No	No	Live/CR (15.2M)
Pt#5	7.2	F	HR	EP300‐ZNF384	2.6M	CR1	positive (0.003%)	IGK2	Undetectable		No	No	Live/CR (21.4M)
Pt#6	0.3	M	HR		12.9M	CR1	Positive (0.029%)	IGK1 and IGK2	Undetectable		No	Yes (5M)	Live/CR (20.1M)
Pt#7	9.6	M	HR		4.7M	CR1	Positive (14.899%)	IGH1 and IGKDE1	Positive (0.975%)	IGKDE1	No	No	Died due to intracranial hemorrhage 4.3M after blina
Pt#8	2.7	M	LR		23.4M	CR1	Positive (0.027%)	IGK1	Undetectable		No	No	Live/CR (12.7M)
Pt#9	4.4	M	IR	E2A‐PBX1	3.2M	CR2	Positive (10.9%)	IGH1	Positive (2.05%)	IGH1	Yes, EM (Periorbital) relapse when blina finished	Yes (3M)	Live/CR (24.9M)
Pt#10	4.1	M	HR		9.0M	CR1	Positive (0.039%)	IGH1, IGK1, and IGK2	Undetectable		No	No	Live/CR (18.0M)
Pt#11	1.8	F	LR		12.3M	CR1	Positive (0.093%)	IGKDE1	Undetectable		No	No	Live/CR (9.6M)
Pt#12	2.9	M	LR		7.5M	CR1	Positive (0.003%)	IGH‐DJ1	Undetectable		No	No	Live/CR (10.5M)
Pt#13	4.8	M	LR	TEL‐AML1	6.8M	CR1	Positive (0.011%)	IGKDE1	Undetectable		No	No	Live/CR (10.2M)
Pt#14	10.6	M	IR	E2A‐PBX1	11.9M	CR1	Positive (0.155%)	IGH1 and IGL1	Positive (0.09%)	IGH1 and IGL1	Yes, bone marrow relapse 1.8 months after blina	Yes (5M)	Live/CR (12.1M)
Pt#15	4.8	M	HR	BCR‐ABL P190	0.9M	CR2	Positive (6.89%)	IGH1	Undetectable		No	Yes (2M)	Live/CR (11.5M)
Pt#16	1.6	M	IR		8.2M	CR1	Positive (0.29%)	IGH1, IGH2, IGKDE1, and IGKDE2	Undetectable		No	No	Live/CR (10.3M)
Pt#17	1.4	F	HR	KMT2A‐USP2	2.6M	CR1	Positive (0.852%)	IGH‐DJ1 and IGH‐DJ2	Undetectable		No	No	Live/CR (12.1M)
Pt#18	3.8	M	IR		4.5M	CR1	Positive (0.093%)	IGH1, IGH3, and IGK1	Positive (0.018%)	IGK1	No	No	Live/CR (13.3M)
Pt#19	8	M	IR		4.7M	CR1	Positive (0.102%)	IGH1, IGK1, and IGL1	Positive (0.051%)	IGK1	No	No	Live/CR (19.5M)

Abbreviations: Blina, blinatumomab; BM, bone marrow; CR, complete remission; CR1, first CR; CR2, second CR; EM, extramedullary; F, female; HR, high risk; IGH, immunoglobulin heavy chain; IGK, immunoglobulin κ light chain gene; IGL, immunoglobulin λ light chain gene; IR, intermediate risk; LR, low risk; M, male; MFC, multiparameter flow cytometry; MRD, minimum residual disease; NGS, next generation sequencing; Pt, patient.

All patients achieved hematological CR with MFC‐MRD level <0.01% before blinatumomab administration, with 16 patients in first CR (CR1) and 3 patients in second CR (CR2). For the three patients in CR2, they all achieved MFC‐MRD remission after VMLD (Vindesine, Mitoxantrone, Peg‐Aspargase, and Dexamethasone) induction. However, the NGS‐MRD was detectable in all patients, with a median level of 0.093% (0.003%–14.899%) (Table 1). As to Ig clones, 19 IGH clones, 4 IGH‐DJ clones, 8 IGK clones, 7 IGKDE clones, and 3 IGL clones persisted even though all of these patients had achieved CR with MFC‐MRD negative status.

Blinatumomab was given 5 μg/m^2^ intravenously daily (not to exceed 9 μg/day) on days 1 through 3 followed by 15 μg/m^2^ daily (not to exceed 28 μg/day) on days 4 through 28. Seven patients were treated for 14 days due to financial problem (5 μg/m^2^ intravenously for 3 days and 15 μg/m^2^ for 11 days, Pt #1, #7, #8, #11, #12, #15, #16). All patients received only one course of blinatumomab.

After blinatumomab consolidation, one patient underwent bone marrow and extramedullary relapse just 1 week after the end of blinatumomab course, while NGS‐MRD turned to be undetectable in 13 patients (68%). The rates of MRD clearance (undetectable) after blinatumomab per NGS were 69% (11/16) and 67% (2/3) in CR1 and CR2 patients, respectively. The clearance rates of IGH, IGK, IGL, IGH‐DJ, and IGKDE after blinatumomab treatment among patients were 75%, 67%, 33%, 100%, and 83%, respectively.

**FIGURE 1:**
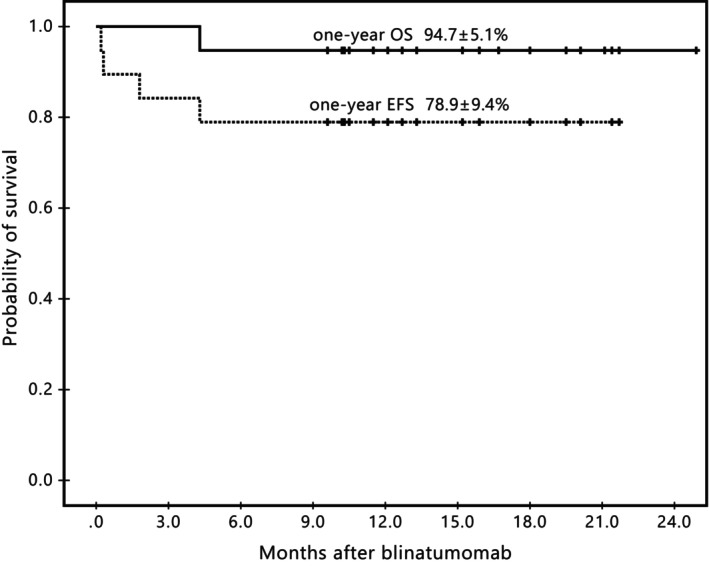
Kaplan–Meier analysis of overall survival (OS) and event‐free survival (EFS). The survival time was calculated from the end of blinatumomab treatment.

Among six cases with persistent MRD after blinatumomab therapy, three patients relapsed from the end of blinatumomab course to 1.8 months later (one with periorbital relapsed at the end of blinatumomab course, one with bone marrow and extramedullary relapse 1 week after blinatumomab, and one with bone marrow relapse 1.8 months later), and all of them underwent allogeneic HSCT and are alive now. All the three patients presented IGH clones resistant after blinatumomab therapy. Pt#2 and Pt#14 were also positive with IGL clones. For the other three patients with persistent NGS‐MRD, two were IGK positive and one was IGKDE positive. All of them underwent ongoing chemotherapy and remained CR at the last follow‐up. This result indicated that IGH seemed more reliable to predict relapse than IGK subtypes. Six patients underwent HSCT after blinatumomab totally. With a median follow‐up of 13.3 months (range 4.3–24.9) from blinatumomab administration, 18 patients were still alive except one patient died from intracranial hemorrhage, with a one‐year overall survival of 94.7% ± 5.1% and event‐free survival of 78.9% ± 9.4% (Figure 1).

## 4 DISCUSSION

Our study observed that patients with bone marrow samples that were considered “undetectable MRD” by MFC had detectable MRD using a highly sensitive NGS‐based MRD assay. We also, for the first time, found that blinatumomab consolidation could rapidly achieve undetectable NGS‐MRD in Chinese pediatric B‐ALL patients.

Measurable residual disease persistence is the most important risk factor for relapse in B‐ALL.^7^ Despite intensive chemotherapy with hematological CR rate of more than 90%, approximately 10%–20% of pediatric patients with ALL in CR exhibit MRD.^8^ In adult B‐ALL patients who failed to achieve MRD responses after at least three blocks of chemotherapy, blinatumomab could induce MRD response rate as high as 80% after only one cycle of treatment.^7^ Furthermore, recent E1910 study in newly diagnosed B‐ALL revealed that adding blinatumomab consolidation in patients with undetectable MFC‐MRD could results in longer survival.^9^ In this study, we also found that blinatumomab could efficiently eradicate MRD and induce deeper molecular remission in patients who were NGS‐MRD positive after chemotherapy, which might indicate a survival benefit.

Recent studies showed that undetectable NGS‐MRD indicated a better prognosis than undetectable MFC‐MRD in B‐ALL and identified future recurrence of relapse.^5^, ^10^, ^11^ Also, a significantly reduced risk of relapse was observed in undetectable NGS‐MRD compared with MRD positivity (5‐year cumulative incidence of relapse: 0% vs. 45%, *p* = 0.04).^12^ Moreover, early NGS‐MRD assessment can identify ALL patients with very low risk of relapse who had excellent long‐term survival.^11^ Thus, National Comprehensive Cancer Network (NCCN) guidelines and consensuses of North American and Canadian experts recommend NGS‐based MRD monitoring for ALL patients.^3^, ^12^, ^13^

In this study, the prognostic significance of different Ig rearrangements varied, with IGH covering most patients and more indicative of relapse, which is consistent with our previous study. Acute lymphoblastic leukemia is usually characterized by a high frequency of unproductive IGH rearrangements caused by constantly active recombinase enzyme and the initiation of IGK/IGL rearrangements that deviate from the allelic exclusion rules due to inappropriate in‐frame selection, which correspond with the findings of our study.^13^ However, the sample size, retrospective design, and short follow‐up period are the limitations of the study. Also, the long‐term survival data was not yet available, so whether the use of blinatumomab impacted patients long‐term outcomes was not certain. Nevertheless, the study adds important clinical information on the prognostic significance of the NGS‐MRD technique in patients with B‐ALL and the therapeutic potential of MRD‐directed blinatumomab in the eradication of MRD in these patients.

Summarizing, with the development of novel and more sensitive MRD detection techniques and advent of new immunotherapies such as blinatumomab, targeting a deeper molecular remission in B‐ALL has now become a reality. Prospective larger studies would be needed to further verify the prognostic value of NGS‐MRD in the era of immunotherapies in ALL.

## AUTHOR CONTRIBUTIONS

**Min'er Gu:** Data curation (lead); formal analysis (equal); investigation (equal); writing – original draft (lead). **Yahong Xia:** Investigation (equal); resources (equal). **Jingying Zhang:** Data curation (equal); investigation (equal). **Yongmin Tang:** Formal analysis (equal); validation (equal); writing – original draft (equal). **Weiqun Xu:** Data curation (equal); investigation (equal). **Hua Song:** Data curation (equal); investigation (equal). **Xiaojun Xu:** Conceptualization (lead); data curation (lead); funding acquisition (lead); investigation (lead); writing – original draft (equal); writing – review and editing (lead).

## FUNDING INFORMATION

This study was supported in part by grants from the Pediatric Leukemia Diagnostic and Therapeutic Technology Research Center of Zhejiang Province (No. JBZX‐201904).

## CONFLICT OF INTEREST STATEMENT

The authors declare that they have no conflict of interests.

## ETHICS STATEMENT

The study was approved by the Ethics Committee of Children's Hospital, Zhejiang University School of Medicine and conducted in accordance with the International Conference on Harmonization Good Clinical Practice guidelines, Declaration of Helsinki and relevant local guidelines and regulations. Informed consents were obtained from parents/guardians of all the enrolled patients.

## CLINICAL TRIAL REGISTRATION

NCT05973032.

## Supporting information


Appendix S1.

## Data Availability

The datasets used and/or analyzed during the current study are available from the corresponding author on reasonable request.
