# Supplementary material for: The effectiveness of blinatumomab in clearing measurable residual disease in pediatric B‐cell acute lymphoblastic leukemia patients detected by next‐generation sequencing
Source: Cancer Med. 2023 Dec 8;12(24):21978–84. doi: 10.1002/cam4.6771 (PMC10757083; doi:10.1002/cam4.6771)

**Supporting Information**

**The effectiveness of blinatumomab on clearing very low level measurable residual disease in pediatric B-ALL patients detected by next-generation sequencing**

**Supplementary Methods**

**Risk stratification of ZJCH-ALL-2019 protocol for newly diagnosed ALL:**

- **Low risk (LR): patients should meet all the following criteria**
  - 1 year ≤ age < 10 years
  - White blood cells < 50 × 10^9^/L
  - Not CNS2, CNSL (CNS3) or / and testicular leukemia
  - Do not have cytogenomic or molecular-biologic characteristics that qualify IR or HR
  - Achieve M1 remission at Day 15 of induction therapy, and keep M1 remission after induction
  - No clinical or image evidence of leukemia involvement after induction, including mediastinal lesion disappearance
  - MRD < 1 × 10^-3^ at Day 15 of induction, MRD < 1 × 10^-4^ after induction (Day 29-33)
- **Intermediate risk (IR): patients should meet at least one of the following criteria**
  - Age ≥ 10 years
  - WBC ≥ 50 × 10^9^/L at diagnosis
  - CNS2, CNSL (CNS3) or / and testicular leukemia
  - t(1;19), i.e. E2A-PBX1
  - T-ALL
  - iAMP21
  - Ph+ ALL
  - Ph-like ALL
  - Mediastinal lesion does not disappear, but shrinks to less than 1/3 of the original size, extramedullary lesions do not total disappear, like splenomegaly, lymphadenopathy, etc.
  - For B-ALL, patients achieve M1 remission on Day 15 of induction; for T-ALL, patients achieve M2 remission on Day 15 of induction; patients should be M1 remission after induction therapy and qualify following MRD criteria
  - On Day 15 of induction, 1 × 10^-3^ ≤ MRD < 1 × 10^-2^; At end of induction (Day 29-33), 1 × 10^-4^ ≤ MRD < 1 × 10^-2^
- **High risk (HR): patients meet one or more of following criteria**
  - For B-ALL, patients achieve M2 remission on Day 15 of induction therapy; for T-ALL, patients achieve M3 remission on Day 15 of induction
  - Patients achieve M2 or M3 remission after induction therapy (Day 29-33)
  - MLL rearrangement
  - Hypodiploid (≤ 44) or DI index < 0.8
  - IKZF1 deletion
  - MEF2D rearrangement
  - TCF3-HLF/t(17;19)(q22;p13)
  - Mediastinal lesion does not shrink to less than 1/3 of the original size
  - Immunophenotype of ETP
  - MRD criteria:
    - For B-ALL, MRD ≥ 1 × 10^-2^ on Day 15 of induction, or MRD ≥ 1 × 10^-2^ after induction (Day 29-33)
    - For T-ALL, MRD ≥ 1 × 10^-2^ after induction (Day 29-33)
    - IgH/TCR clonal analysis shows MRD ≥ 1 × 10^-4^ until Week 12

Notes:

M1: bone marrow blasts < 5%

M2: 5% ≤ bone marrow blasts < 20%

M3: 20% ≤ bone marrow blasts

**ZJCH-ALL protocol:**

| **Regimens** | **LR** | **IR** | | **HR** |
| --- | --- | --- | --- | --- |
| **Induction** | VDLD + PEG-ASP × 2 + IT × 2 | VDLD + PEG-ASP × 2 + IT × 4 | | VDLD + PEG-ASP × 2 + IT × 4 |
| **Consolidation** | Mini CAM × 1, d1 IT | B-ALL:  CAM × 1, d1 IT | T-ALL:  CAM × 1, d1 IT, plus (HD-MTX + 6-MP) × 2, CAML | CAM × 1, d1 IT, plus (HD-MTX + 6-MP) × 2, CAML |
| **Extramedullary prophylaxis** | (HD-MTX 3 g/m + 6-MP, d1 IT) × 3 | (HD-MTX 5 g/m + 6-MP, d1 IT) × 3 | (HD-MTX 5 g/m + 6-MP, d1 IT) × 1 | (HD-MTX 5 g/m + 6-MP, d1 IT) × 1 |
| **Early intensive treatment** | VDLD (anthracycline × 2, PEG-ASP × 2) | VDLD (anthracycline × 2, PEG-ASP × 2) + VALD | | VDLD (anthracycline × 2, PEG-ASP × 2) + VALD |
| **Maintenance** | 6-MP + MTX + VD repeating | 6-MP + MTX + VD repeating | | 6-MP + MTX + VD repeating, COAD in the 3^rd^ and 9^th^ months every year after patients achieve complete remission (CR) |
| **Regular intensive treatment** | VALD + miniCAM half a year, VDLD one year, and COAD one and a half years after patients achieve CR | VALD + CAM half a year after patients achieve CR, VDLD at 1.5 years post CR, VALD after one and two years of CR | | VALD + CAM half a year after patients achieve CR, VDLD at 1.5 and 2.5 years after CR, VALD after one and two years of CR |
| **Following extramedullary prophylaxis** | HD-MTX (3 g/m) × 2 after regular intensive treatment every half a year, total five cycles | HD-MTX (5 g/m) × 2 after regular intensive treatment every half a year, total six cycles. For patients with original CNSL or testicular leukemia, increase to 9 cycles | | HD-MTX (5 g/m) × 2 after regular intensive treatment every half a year, total seven cycles. For patients with original CNSL or testicular leukemia, increase to 9 cycles |
| **Treatment duration** | 2 years | 2 years | | 2.5 years |
| IT: lumbar puncture + intrathecal injection (MTX, Ara-C, DXM) | | | | |

**Methods**

**Sample preparation**

Bone marrow (BM) aspirations were collected at baseline before blinatumomab and after blinatumomab treatment. BM was cryopreserved and stored at -80^o^C until further use. // BM aspirations were immediately processed for DNA extraction.

**DNA extraction**

Genomic DNA was isolated from bone marrow samples following standard protocol by using the QIAamp DNA Mini kit (Qiagen, Germany) and its instructions. The input DNA for diagnostic samples (clonality test) was 500 ng and for post-treatment samples (MRD tracking test) is at least 7μg which corresponds to 1x10^6^ mononuclear cells (MNCs).

**Quantitative PCR/Multiplex PCR**

The extracted genomic DNA was amplified using locus-specific primer sets for the immunoglobulin heavy-chain locus complete IGH (23 primers), incomplete IGH (9 primers), immunoglobulin kappa locus (IGK) (15 primers, KDE included) and immunoglobulin lambda locus (IGL) (17 primers) in the first PCR reaction. Twenty-five cycles of PCR amplification were carried out by 3 tubes of multiplex PCR (Qiagen, Germany) under conditions of 30 seconds at 94°C, 90 seconds at 58°C, and 30 seconds at 72°C, plus a final extension for 10 minutes at 72°C. The goal of this amplification reaction was to reproducibly amplify all possible rearranged sequences in the samples while appending the housekeeping reference. Next, 1:50 of these PCR products was further amplified in a second PCR reaction of eighteen cycles carried out by 200 units of PrimeSTAR HS DNA Polymerase (Takara, Japan) under conditions of 30 seconds at 94°C, 30 seconds at 58°C, and 30 seconds at 72°C, plus a final extension for 10 minutes at 72°C, using universal primers complementary to the adaptors that were linked to the locus-specific primers with sample-identifiers. Multiple libraries were purified by AMPure XP beads (Beckman, America) and pooled into one library.

The first-step PCR primers were aimed to acquire all the V(D)J rearrangement gene segments of the Ig at the genomic DNA level. There are three sets of locus-specific primers covered from the FR1/FR2/FR3 V regions to J regions, that will reduce the probability of amplification failure due to somatic mutations or genetic mutations. Subsequently, universal sequencing primers with independent labels were extended to both ends of the Ig libraries in the second-step PCR. The combination of primers according to Illumina® sequencing platform produced a unique index to for each library for the next step in bioinformatics analysis. The pooled library was subjected to sequencing using the Illumina® Novaseq PE150 platform (v), and the sequences and frequencies of the different clonotypes in the samples were obtained.

**Supplementary Figures S1.** Flow chart of patients included in this study.


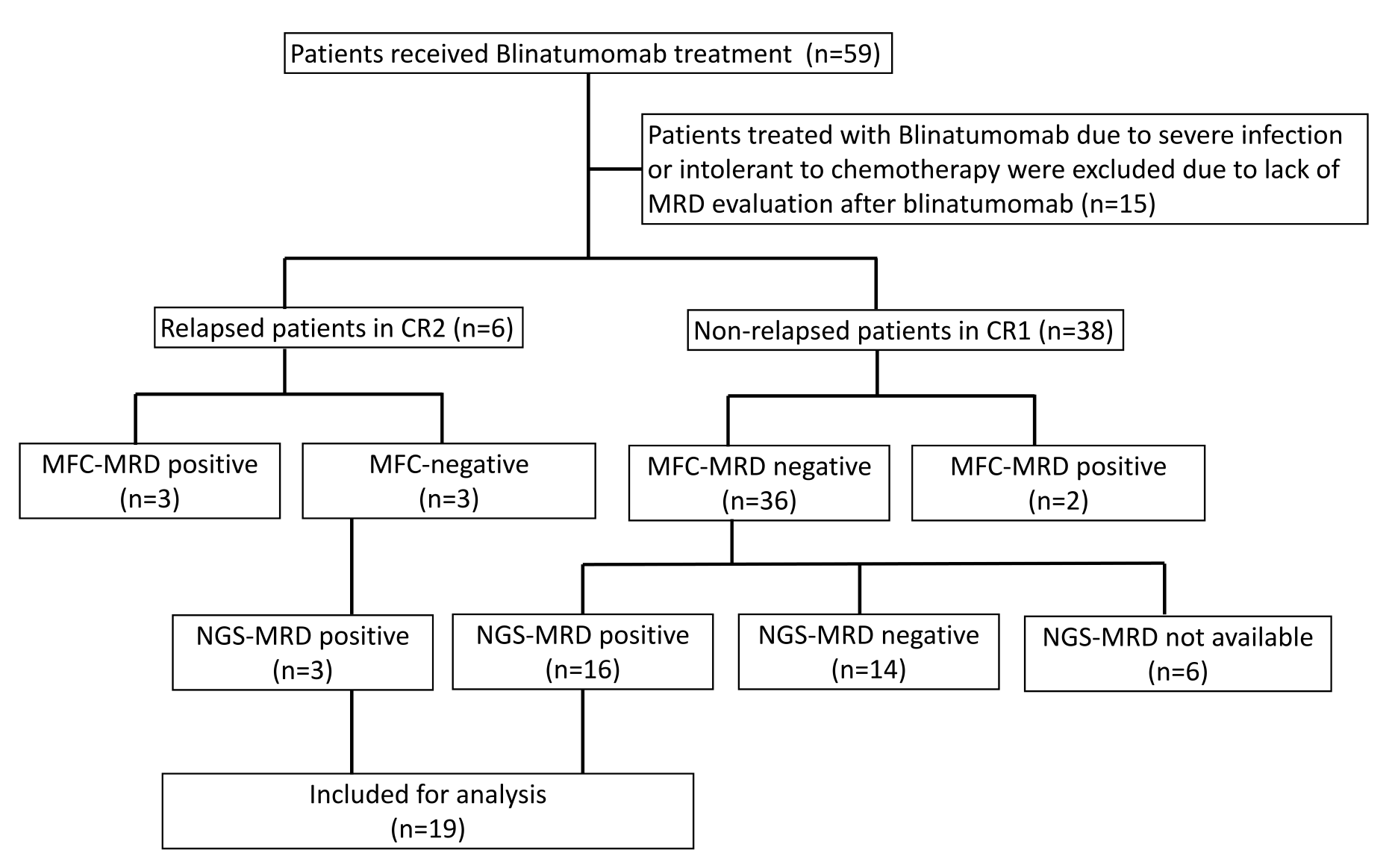

Supplement: Supplementary file 1 — Appendix S1. [file CAM4-12-21978-s001.docx]
